# Irisin alleviated sepsis via enhancing macrophage phagocytosis and reducing inflammation levels

**DOI:** 10.3389/fimmu.2025.1618699

**Published:** 2025-08-15

**Authors:** Zeen Gong, Yunyan Ji, Haiyang Wu, Linli Xue, Xiuju Yu, Xiaomao Luo, Yi Yan, Jiayin Lu, Juan Wang, Yanjun Dong, Haidong Wang

**Affiliations:** ^1^ College of Veterinary Medicine, Shanxi Agricultural University, Jinzhong, Shanxi, China; ^2^ Department of Nephrology, Shanghai General Hospital, Shanghai Jiao Tong University School of Medicine, Shanghai, China; ^3^ College of Veterinary Medicine, China Agricultural University, Beijing, China

**Keywords:** irisin, sepsis, macrophage, phagocytosis, inflammation

## Abstract

**Background:**

The treatment of sepsis relies on antibiotics following infection; however, the emergence of resistant bacteria necessitates the development of new therapeutic agents. Irisin has been shown to alleviate symptoms in septic mice, although its mechanism of action remains unclear. Our aim was to determine the mechanism by which irisin alleviates sepsis.

**Methods:**

In this study, septicemia was induced in mice using *Escherichia coli* CMCC 44102 (E) and *Staphylococcus aureus* CMCC 26003 (SA). The level of serum irisin were examined by ELISA kit. Mice with septicemia were intraperitoneally injected with irisin. The survival rate, body temperature, clinical manifestations, body’s bacterial load, the number of immune cells in blood, the level of inflammation in the body of the mice with septicemia were monitored to evaluate the effect of irisin therapy. The effect of irisin on the phagocytosis of spleen macrophages was observed by flow cytometry. LPS was used to induce inflammation in RAW264.7 cells and irisin was added to determine the effect of irisin on the level of macrophage inflammation.

**Results:**

*In vivo*, sepsis decreased serum irisin levels in mice. Irisin treatment in septicemic mice enhanced the phagocytosis of splenic macrophages, improved survival rates, accelerated body temperature recovery, alleviated clinical symptoms, reduced serum and organ inflammation, lowered bacterial loads in organs and body fluids. *In vitro*, irisin increased phagocytosis and reduced inflammation in RAW264.7 cells.

**Conclusions:**

Irisin improves splenic macrophage phagocytosis and reduces macrophage inflammation, thereby mitigating sepsis. Irisin holds potential as a therapeutic agent for sepsis treatment.

## Introduction

Sepsis, a life-threatening organ dysfunction caused by dysregulated host responses to infection (1), affected 48.9 million patients globally in 2017 with 22.5% mortality (2). It arises when pathogens breach barrier tissues or commensal microbiota disrupt these barriers (3–5). For example, *Escherichia coli* in feline/canine luteal phases causes uterine pyometra (6, 7); its bloodstream invasion releases endotoxins inducing fever, lethargy, multi-organ infections, and fatal sepsis (8, 9). *Escherichia coli* is a primary etiological agent of Gram-negative septicemia (10). Conversely, *Staphylococcus aureus* initiates skin/soft tissue infections, migrating from wounds to deep tissues and circulation causing sepsis (11, 12), thus representing a major Gram-positive septicemia pathogen (13). Current post-infection antibiotic therapies (14) underscore the critical need for alternative treatments to combat antibiotic resistance.

The spleen serves as a crucial peripheral immune organ within the body, storing a significant number of mature immune cells. Splenic macrophages are capable of phagocytosing bacteria present in the bloodstream, thereby making the spleen a focal point in sepsis research. In patients who have undergone splenectomy, the capacity of monocytes to phagocytose and kill Candida albicans is markedly diminished, resulting in an elevated risk of sepsis (15). In a separate study, it was observed that Salmonella bound to lymphocytes in the peripheral blood of typhoid patients was phagocytized by splenic macrophages (16). Following splenectomy in dogs, the absence of phagocytic activity led to a reduction in endotoxin clearance (17). These findings collectively indicate that the spleen plays a direct role in the clearance of bacteria associated with sepsis. Additionally, macrophages are integral to the body’s inflammatory response during sepsis. Lipopolysaccharide (LPS) stimulates macrophages to adopt an M1 phenotype, characterized by potent pro-inflammatory, phagocytic, and antigen-presenting abilities. However, excessive activation of M1 macrophages can result in severe inflammation and tissue damage (18). Conversely, Interleukin-4 (IL-4) promotes the differentiation of macrophages into the M2 phenotype, which exhibits anti-inflammatory properties and supports angiogenesis and tissue repair (18). Moreover, the interplay between pro-inflammatory and anti-inflammatory macrophage phenotypes significantly influences the progression of both ovarian cancer (19) and osteosarcoma (20), suggesting their potential involvement in sepsis pathogenesis.

Irisin is a small protein primarily secreted by muscle tissue following exercise, formed through the truncation of the secreted protein fibronectin type III domain-containing protein 5 (FNDC5) at glutamate 112 (excluding the signal sequence) (21). Various tissues and organs—including the myocardium, white adipose tissue, thyroid, adrenal gland, central nervous system, and liver—also synthesize and release irisin (22–26). This protein facilitates the browning of white adipose tissue (21), enhances memory in mice (27), promotes the thickness of bone trabeculae (28), increases cardiomyocyte proliferation (29), raises reproductive hormone levels (30), and diminishes macrophage inflammation whereas regulating the intensity of the macrophage respiratory burst (31, 32). Recent research has indicated a reduction in serum irisin levels during septic cases (33), suggesting its potential involvement in the pathophysiology of sepsis.

The present study observed a decreased level of irisin in serum samples from mice infected with E and SA to model sepsis. Administration of intraperitoneal irisin in septic mice resulted in improved survival rates and alleviated symptoms. Subsequently, mice were infected with the *Escherichia coli* DH5α strain tagged with green fluorescent protein (EGFP) to simulate sepsis, followed by an intraperitoneal injection of irisin. Flow cytometric analysis demonstrated that irisin enhanced the phagocytic function of splenic macrophages. *In vitro* experiments revealed that irisin improved the proliferation and phagocytic activity of RAW264.7 cells and reduced LPS-induced inflammation in these cells. Our findings may provide a novel therapeutic approach for the management of sepsis.

## Materials and methods

### Bacterial strains and plasmid

E and SA were obtained from the National Center for Medical Culture Collections (CMCC). Both strains were cultivated in Luria-Bertani (LB) medium at 37°C. Plasmid pGFPuv (Novopro, V012009) was transformed into E DH-5α (Takara Bio, 9057) following the manufacturer’s protocol. Briefly, 6 ng of plasmid pGFPuv was added to melted DH-5α cells, gently mixed, and subsequently placed on ice for 30 min. The tube containing DH-5α was then incubated in a 42°C water bath for 90 s, followed by an ice bath for 180 s. An additional 800 μL of LB medium was added to the tube, which was then cultured at 37°C and 200 rpm for 1 hour before being centrifuged at 4000 rpm for 5 min. The DH-5α strain carrying pGFPuv was inoculated onto LB agar containing ampicillin (A^+^, 100 mg/mL) and cultured overnight at 37°C. A single colony was then selected and subsequently cultured in A^+^LB medium at 200 rpm and 37°C.

### Mice

Six-week-old female BALB/c mice were acclimatized for one week in a specific pathogen-free experimental animal facility before being utilized for bacterial infection and irisin treatment models.

### Murine infection and treatment model

#### Preparation of bacteria and irisin

E, SA, and EGFP were cultured in LB medium at 37°C until the optical density at 600 nm (OD_600_) reached 0.6-0.8. Bacterial cells were then washed and resuspended in sterile PBS prior to use.

Building upon prior methodologies, we established murine sepsis models with optimized protocols (34). Prior to infection, mice had free access to water and were fasted for 12 h. The mice were randomly divided into three groups, each containing 12 animals. E was intraperitoneally injected into each group at dosages of 8×10^7^ CFU/mL, 8×10^8^ CFU/mL, and 8×10^9^ CFU/mL, respectively (200 μL per mouse). The survival rates of the mice in each group were monitored over 48 h to determine the moderate dose (8×10^8^ CFU/mL) of E. An additional 36 mice were randomly assigned to three groups, each receiving an intraperitoneal injection of 8×10^8^ CFU/mL of E in 200 μL. Each group subsequently received intraperitoneal injections of irisin (Phoenix Pharmaceuticals, 067-17, USA) at doses of 0.5 μg/g (body weight), 5 μg/g, and 50 μg/g, administered 3 h before and 0.5 h after bacterial infection. The survival rates of the mice were observed over 48 h to establish the optimal dose of irisin (5 μg/g).

Dose-response studies were also conducted for SA (n=10 mice per group) and EGFP (n=12 mice per group) analogously to E. The moderate dose for SA was determined to be 5×10^10^ CFU/mL (200 μL per mouse), whereas the moderate dose for EGFP was found to be 1×10^9^ CFU/mL (200 μL per mouse). The optimal dose of irisin was consistently 5 μg/g for both SA and EGFP.

12 mice were divided evenly into three groups (n=4), The control group received an intraperitoneal injection sterile PBS of 200 µL. The E group was administered an intraperitoneal injection of 200 µL of 8×10^8^ CFU/mL of E. The SA group was administered an intraperitoneal injection of 200 µL of 5×10^10^ CFU/mL of SA. After 12 h, the mice’s blood was collected. Serum irisin levels were measured with an irisin enzyme-linked immunosorbent assay (ELISA) kits (Elabscience, E-EL-M2743, China).

#### Evaluation of the therapeutic effect of irisin

Mice were randomly assigned to three groups, with each group containing 12 mice (n=12). The control group received an intraperitoneal injection sterile PBS of 200 µL. The E group was administered an intraperitoneal injection of 200 µL of 8×10^8^ CFU/mL of E. In the *Escherichia coli* CMCC 44102+irisin (EI) group, mice were given an intraperitoneal injection of 200 µL of 8×10^8^ CFU/mL of E, along with an additional intraperitoneal injection of 5 μg/g irisin administered 3 h prior to bacterial infection and again 0.5 h following the infection. To monitor the effects of the bacterial infection over time, the anal temperature of the mice was recorded at intervals of 0, 1, 2, 3, 6, 12, 24, 36, and 48 h. Furthermore, the appearance, behavior, clinical characteristics, and hydration status of the mice were assessed based on the clinical criteria outlined by Yin (34) (**Supplementary Table S2**). At 3, 6, and 12 h post-bacterial infection, three mice from each group were randomly euthanized, and samples of blood, peritoneal washes, and tissues (heart, liver, spleen, lung, kidney) were collected. Routine blood tests were conducted on the blood samples. Serum was extracted, and the levels of interleukin-1β (IL-1β) (Jianglaibio, JL18442, China) and interleukin-6 (IL-6) (Jianglaibio, JL20268, China) in the serum were quantified using ELISA kits according to the manufacturer’s instructions. The bacterial load in the mice was assessed using Yin’s method (34), which involved serially diluting the blood, peritoneal washes, and tissues, followed by applying 10 µL of the sample onto LB agar plates. These plates were incubated at 37°C overnight, and colony-forming unit (CFU) counts were subsequently performed. Total RNA was extracted from the tissues, and the transcription levels of *IL-1β*, *IL-6*, and *tumor necrosis factor-α* (*TNF-α*) were measured using quantitative reverse transcriptase polymerase chain reaction (qRT-PCR).

Similar experiments were conducted with SA (n=10 mice per group). All procedures were identical, with the exception that the dose of SA was 5×10^10^ CFU/mL.

### Flow cytometry

24 mice were randomly divided into two groups. The EGFP group received an intraperitoneal injection of 200 µL of 1×10^9^ CFU/mL EGFP. In the *Escherichia coli* DH5α strain tagged with green fluorescent protein+irisin (EGFPI) group, mice were intraperitoneally injected with 200 µL of 1×10^9^ CFU/mL EGFP, along with 5 μg/g irisin administered 3 h prior to infection and 0.5 h after infection. Twenty-four h following bacterial infection, three mice from each group were randomly euthanized, and spleen samples were collected for flow cytometry analysis.

The spleens were ground as thoroughly as possible. Following this, 7 mL of HBSS solution—containing 1% FBS, 2.5 U/mL collagenase type IV (Worthington, LS004188, USA), and 10 mM CaCl_2_—was added, and the mixture was digested at 37°C, 120 rpm, for 30 min. To terminate the digestion, 1 mM EDTA was incorporated into the mixture and allowed to react for 5 min at room temperature. The resulting mixture was then passed through a 70 μm cell filter and collected in a centrifuge tube. Subsequently, the mixture was centrifuged at 4°C at 300 g for 5 min, and the supernatant was discarded. Following this, 1× red blood cell lysate (Solarbio, R1010, China), pre-cooled to 4°C, was added to the tube, and the precipitate was fully suspended and placed on ice for 5 min. The suspension was washed with cold PBS and centrifuged at 300 g, 4°C for another 5 min. Finally, the supernatant was discarded, and HBSS solution (containing 1% FBS) was used to resuspend the cells, achieving a cell concentration of 1×10^7^ cells/mL. Cell data were collected using a BD FACSCanto II flow cytometer and analyzed with FlowJo 10.0.7 software (Tree Star, USA). The antibodies utilized in this study included anti-mouse/human CD11b-APC (BioLegend, 101211, USA), anti-mouse Ly6G-PE-Cy7 (BioLegend, 127617, USA), anti-mouse CD45-Percp (BioLegend, 103129, USA), anti-mouse F4/80-APC-Cy7 (BioLegend, 123117, USA), and anti-mouse CD3e-PE (BioLegend, 100307, USA), all applied in accordance with the manufacturer’s instructions.

### Cell culture

RAW264.7 cells (ATCC, TIB-71) were cultured in high-glucose Dulbecco*’*s Modified Eagle*’*s Medium (DMEM) supplemented with 10% fetal bovine serum (FBS) and 1% streptomycin/penicillin at 37°C with 5% CO_2_.

### Phagocytosis assay

RAW 264.7 cells were seeded in 24-well plates at a concentration of 1×10^6^ cells per well and cultured for 6 h to facilitate cell attachment. EGFP was washed once with sterile PBS and then resuspended in DMEM supplemented with 10% FBS. Bacteria were co-cultured with RAW 264.7 cells at a multiplicity of infection (MOI) of 1 (1×10^6^ cells, 1×10^6^ bacteria per well), with 20 nM irisin added to 6 wells. The co-culture was maintained at 37°C with 5% CO_2_ for 1 hour, whereas 6 wells without irisin served as the negative control. Next, 0.15 mL of streptomycin/penicillin per mL of medium was added to the wells, and the cells were cultured at 37°C with 5% CO_2_ for an additional hour to kill the extracellular bacteria. Finally, the cells were washed with sterile PBS three times.

In the three-well negative control group and the three-well irisin-supplemented RAW264.7 cells, 1 mL of 4% paraformaldehyde was added, and the cells were fixed at room temperature for 20 min. The cells were then washed with sterile PBS three times. Subsequently, a sufficient amount of DAPI was added to the wells, and the cells were incubated at room temperature for 20 min. Finally, the liquid in the wells was discarded, and the cells were washed with PBS four times. Observation of the cells was conducted using a laser confocal microscope (Olympus, FV1000, Japan).

0.5 mL of a 1% (v/v) Triton X-100 solution was added to each well of the three-well negative control group and the three-well irisin-supplemented RAW264.7 cells. After 10 min, 100 µL of gradiently diluted lysate was coated onto A^+^LB agar and cultured at 37°C overnight to count the number of live bacteria.

### Immunofluorescence

RAW264.7 cells were cultured to subfusion, the cells continued to be cultured in complete medium containing 20 nM irisin/mL for 30 min. The cells were then fixed with 1.5 mL of 4% paraformaldehyde at room temperature for 30 min. Following fixation, the cells were washed with sterile PBS three times and subsequently blocked with 10% goat serum at room temperature for 35 min. The cells were incubated overnight at 4°C with primary antibodies, specifically anti-irisin (1:100, Phoenix Pharmaceuticals, H-067-17, Rabbit) and anti- integrin αV (ITG αV) (1:50, Santa Cruz, SC-376156, Mouse). After primary antibody incubation, the cells were washed with PBS three times. The cells were then incubated with fluorescent secondary antibodies (488 goat anti-rabbit fluorescent secondary antibody 1:200, 594 goat anti-mouse fluorescent secondary antibody 1:200) at 37°C for 1 hour, followed by three washes with PBS. Finally, the cells were incubated with DAPI at room temperature for 20 min, washed with PBS four times, and observed under a confocal laser microscope.

### Assessment of cell proliferative activity

After RAW264.7 cells reached subconfluence, they were resuspended and seeded in 96-well plates (100 µL/well) and cultured at 37°C with 5% CO_2_ for 6 h. Following the change to a new complete medium, varying concentrations of irisin (0 nM, 10 nM, 20 nM, 40 nM, 80 nM, 160 nM) were added to each well (with each concentration repeated six times), and the cells were cultured for an additional 12 h. Subsequently, 10 µL of CCK-8 solution (YEASE, 40203ES80, China) was added to each well, and the cells were cultured for another 2 h. The absorbance of each well at 450 nm was measured, and cell proliferation activity was calculated according to the manufacturer’s instructions. Furthermore, the cells were seeded again in 96-well plates (100 µL/well) and cultured at 37°C with 5% CO_2_ for 6 h. After this, the complete medium was replaced, and 20 nM irisin was added to the wells, with cells cultured for 0h, 6h, 12h, 24h, and 48h (four repeats for each time point). Subsequently, 10 µL of CCK-8 solution was added to each well, and the cells were cultured for an additional 2 h. The absorbance of each well at 450 nm was measured, and cell proliferation activity was calculated as specified by the manufacturer’s instructions.

### LPS stimulated RAW264.7 cells

RAW264.7 cells were seeded in 6-well plates and cultured to subconfluence. LPS (Sigma, L6529, USA) (100 ng/mL) was added to each well of the LPS (L) group; LPS (100 ng/mL) and irisin (20 nM) were added to each well of the LPS+irisin (LI) group; irisin (20 nM) was added to each well of the irisin (I) group; and an equivalent volume of PBS was added to each well of the control (C) group. Each treatment was repeated three times. The cells were then cultured at 37°C with 5% CO_2_ for 12 h.

### qRT-PCR analysis

Total RNA was extracted using RNAiso Plus (Takara, 9109, Japan) and subsequently utilized for cDNA synthesis with a reverse transcription kit (Vazyme, R223-01, China). The primers used in this study were designed with Primer 5 software and synthesized by Shanghai General Biological Company (China). The sequence information is presented in **Supplementary Table S6**. Following the manufacturer’s instructions, a mixture of cDNA, primers, and SYBR was prepared and placed in the QS5 system, using the following thermal cycling program: 95°C for 30 s; 95°C for 10 s; 60°C for 30 s (40 cycles); 95°C for 15 s; 60°C for 1 minute; and finally, 95°C for 1 second. The results were expressed as ^ΔΔ^CT values.

### Western blot

RAW264.7 cells were cultured to subconfluence. RIPA buffer (Beyotime, P0011, China) and PMSF were employed for the extraction of total protein from the cells. The total protein concentration was determined using a BCA kit (Beyotime, China). Following SDS-PAGE, the proteins were transferred to a PVDF membrane (Millipore, ISEQ00010, USA). The PVDF membrane was then blocked with a 5% skim milk powder solution at 37°C for 1 hour. Subsequently, the membrane was incubated with primary antibodies overnight at 4°C, followed by incubation with a HRP-conjugated secondary antibody (anti-rabbit IgG, 1:20,000; CWBIO, CW0156S, China) at 37°C for 1 hour. The primary antibodies utilized in this study included anti-FNDC5 (1:1000; Abcam, ab-174833; Rabbit) and anti-irisin (1:1000, Phoenix Pharmaceuticals, G-067-17, Rabbit). Target bands were visualized using the enhanced ECL kit (CWBIO, CW0049S, China).

### Statistical analysis

Statistical results are expressed as mean ± standard error of mean (SEM). Differences between data sets were analyzed using a two-tailed unpaired t-test, with p< 0.05 considered statistically significant. All data visualizations were performed using GraphPad Prism 9.5 software.

## Result

### Irisin deficiency and survival rescue in murine sepsis

**Figure 1:**
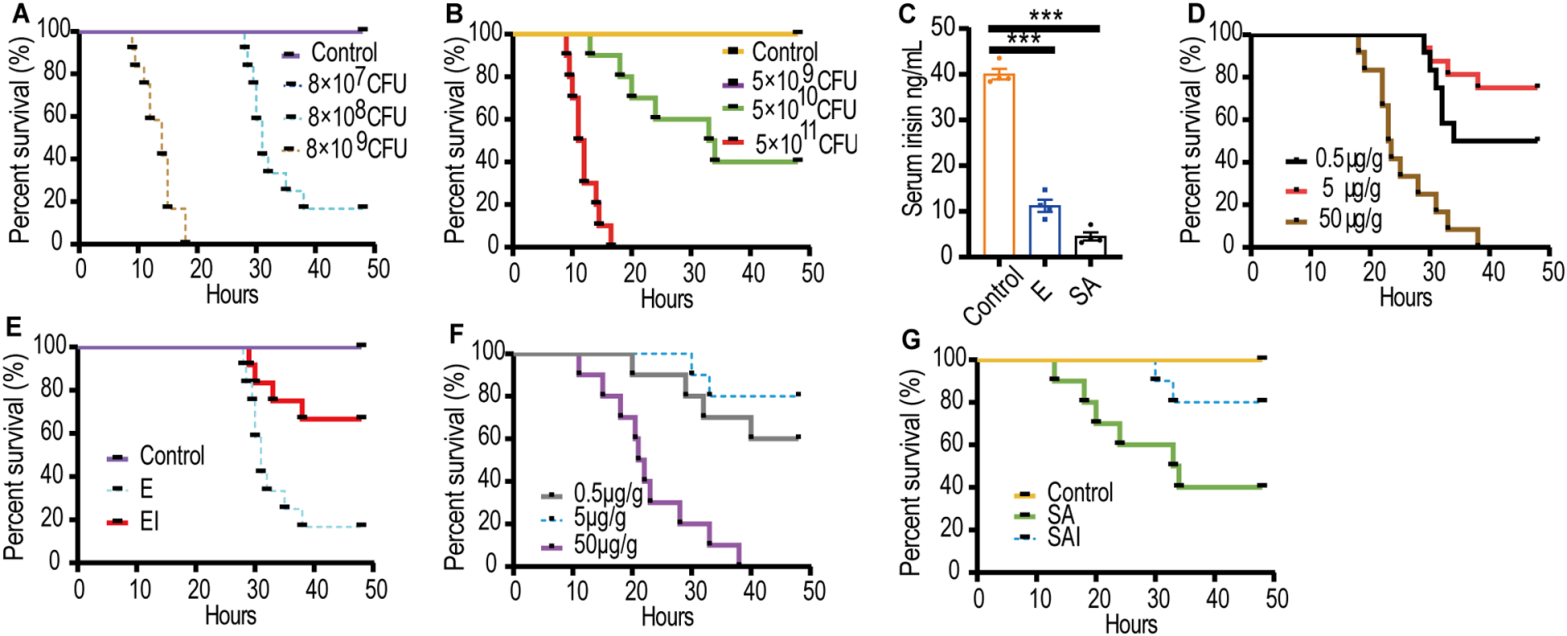
Serum irisin levels reduced in septic mice models. **(A)** Optimal dose of E to cause sepsis in mice, n=12/group. **(B)** Optimal dose of SA to cause sepsis in mice, n=10/group. **(C)** Serum irisin levels in mice with septicemia induced by E, SA and normal mice. n=4/group. **(D)** Optimal dose of irisin for relieving E sepsis. n=12/group. **(E)** Irisin improved the survival rate of mice with E sepsis. n=12/group. **(F)** Optimal dose of irisin for relieving SA sepsis. n=10/group. **(G)** Irisin improved the survival rate of mice with SA sepsis. n=10/group. Mean ± SEM, ***P < 0.001.

Septic mouse models with excessively strong or weak virulence were deemed unsuitable for further experiments. Consequently, an E septic mouse model was established using 8×10^8^ CFU/mL of E, which resulted in a survival rate of 16.7% (**Figure 1A**). Similarly, a SA septic mouse model was developed using 5×10^10^ CFU/mL of SA, yielding a survival rate of 40% (**Figure 1B**). Serum irisin levels in the E septic mouse model (11.24 ± 1.33 ng/mL) and the SA septic mouse model (4.52 ± 0.87 ng/mL) were significantly lower than those in healthy mice (40.02 ± 1.17 ng/mL), as shown in **Figure 1C**.

We hypothesized that irisin treatment would decrease the mortality of septic mice. Accordingly, irisin doses of 0.5 μg/g, 5 μg/g, and 50 μg/g were injected intraperitoneally into septic mice to identify the optimal therapeutic dose. In the E septic mouse model, the optimum therapeutic dose was determined to be 5 μg/g, which improved the survival rate from 16.7% to 66.7% (**Figures 1D, E**). Similarly, in the SA septic mouse model, 5 μg/g of irisin was also found to be the optimal therapeutic dose, increasing the survival rate from 40% to 80% (**Figures 1F, G**).

#### Irisin alleviated symptoms in E septic mice

**Figure 2:**
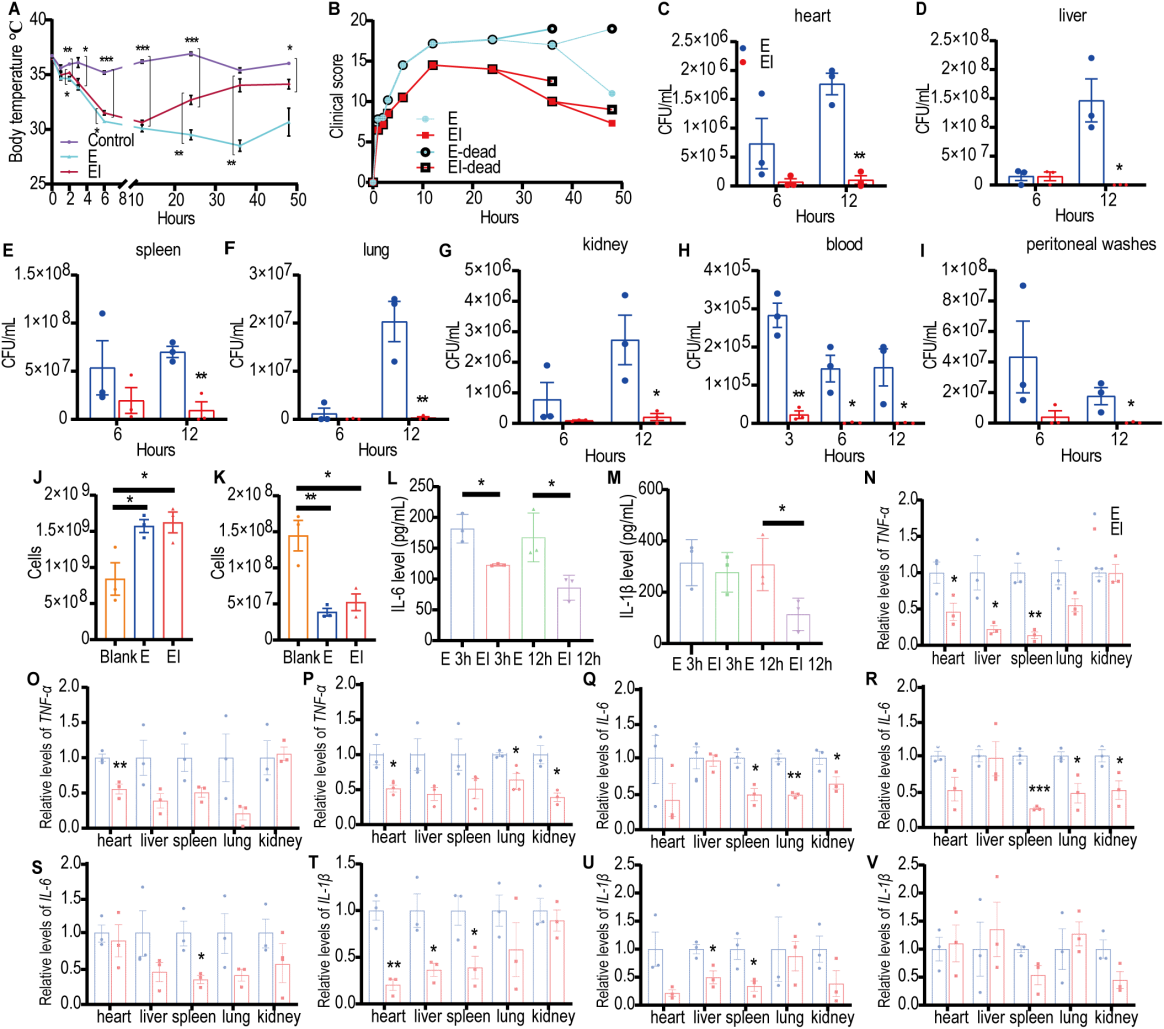
Irisin improved the performance of mice with E induced septicemia. **(A)** Irisin increased body temperature in mice with E sepsis. At each time point, the temperature of three mice in each group was measured. **(B)** Clinical manifestations of mice with E sepsis improved by irisin. At each time point, data were collected from three mice in each group. **(C-I)** Bacterial load in organs (heart, liver, spleen, lung, kidney) and body fluids (blood and peritoneal washes) of mice with E septicemia. n=3/group/time point. **(J)** Neutrophils in the blood of mice with septicemia induced by **(E)** n=3/group. **(K)** Monocytes in the blood of mice with septicemia induced by **(E)** n=3/group. **(L)** Serum IL-6 levels in mice with E induced sepsis. n=3/group/time point. **(M)** Serum IL-1β levels in mice with E induced sepsis. n=3/group/time point. **(N-P)** *TNF-α* mRNA levels in organs of mice at 3h, 6h and 12h of E induced sepsis. n=3/group. **(Q-S)** *IL-6* mRNA levels in organs of mice at 3h, 6h and 12h of E induced sepsis. n=3/group. **(T-V)** *IL-1β* mRNA levels in organs of mice at 3h, 6h and 12h of E induced sepsis. n=3/group. Mean ± SEM, *P<0.05, **P<0.01, ***P<0.001.

To investigate the mechanism by which irisin improves the survival rates of mice with E sepsis, BALB/c mice were challenged with 200 µL of E at 8×10^8^ CFU/mL, with or without the intraperitoneal injection of 5 μg/g of irisin. As shown in **Figure 2A** and **Supplementary Table S1**, the body temperature of the control group remained stable within the normal range throughout the 48h experiment. In the *Escherichia coli* CMCC 44102+irisin (EI) group, body temperature decreased from 36.46 ± 0.17°C to 30.63 ± 0.20°C over the first 12 h, then increased from 30.63 ± 0.20°C to 34.13 ± 0.44°C between 12 and 48 h. In contrast, the body temperature of the E group also decreased from 36.73 ± 0.14°C to 28.5 ± 0.51°C during the first 36 h, subsequently rising from 28.5 ± 0.51°C to 30.66 ± 1.27°C from 36 to 48 h. The body temperature of the EI group remained intermediate between the control and E group throughout the 48 h period.

We also assessed the impact of irisin on clinical manifestations, including appearance, behavior, respiratory characteristics, and hydration status, in E septic mice and recorded the results. As indicated in **Figure 2B** and **Supplementary Table S3**, the average clinical scores of the E group consistently increased after the E challenge, peaking at 17.67 at 24 h, before progressively decreasing to 11.00 at 48 h. In the EI group, clinical scores similarly rose after the E challenge, reaching a maximum of 14.50 at 12 h, and then declining to 7.25 at 48 h. At each time point throughout the study, the E group exhibited higher clinical scores than the EI group.

To investigate the level of bacterial load in E septic mice, we conducted bacterial culture analyses on organ homogenates and body fluids obtained from the mice. As illustrated in **Figures 2C–I** and **Supplementary Figures S1A–C**, 12 h post-bacterial infection, the viable bacterial count in the E group was significantly higher than in the EI group across the heart, liver, spleen, lung, and kidney. In the blood, the viable bacterial count in the E group was notably greater than that in the EI group at 3, 6, and 12 h following the bacterial attack. Additionally, in the peritoneal flushing solution, the viable bacterial count in the E group was significantly higher than that in the EI group at 12 h post-infection. These findings suggest that irisin treatment may effectively reduce bacterial load in E septic mice.

Once bacteria invaded the body, they disseminated to tissues and organs via the bloodstream, ultimately resulting in sepsis. Therefore, blood tests serve as an important indicator of sepsis progression. The results of blood neutrophil (NEU) detection indicated that the number of NEU in both the E and EI groups was significantly higher than in the control group; however, there was no significant difference between the E and EI groups (**Figure 2J**). Similarly, as shown in **Figure 2K**, blood monocyte (MON) detection revealed that the number of MON was significantly elevated in both the E and EI groups compared to the control group, with no significant difference observed between these two groups. Further analysis of pro-inflammatory factors IL-6 and IL-1β in serum showed that the IL-6 levels in the EI group were significantly lower than in the E group at 3 h and 12 h post-bacterial infection (**Figure 2L**). The IL-1β levels in the EI group were also significantly lower than in the E group at 12 h after infection, although no significant difference was noted at the 3 h mark (**Figure 2M**). Although irisin did not appear to regulate the counts of NEU and MON in the blood, it did reduce the levels of inflammatory factors in serum, suggesting that irisin plays a role in the development of E sepsis.

We also evaluated the effects of irisin on the transcription levels of pro-inflammatory factors *TNF-α*, *IL-6*, and *IL-1β* in various organs of E septic mice. As shown in **Figures 2N–V**, at least one pro-inflammatory transcription factor in the EI group was significantly downregulated in the heart, liver, spleen, lung, and kidney within 12 h after bacterial infection (with the exception of the liver at 12 h). These results suggest that irisin reduces inflammation levels in the organs of E septic mice.

#### Irisin alleviated symptoms in SA septic mice

**Figure 3:**
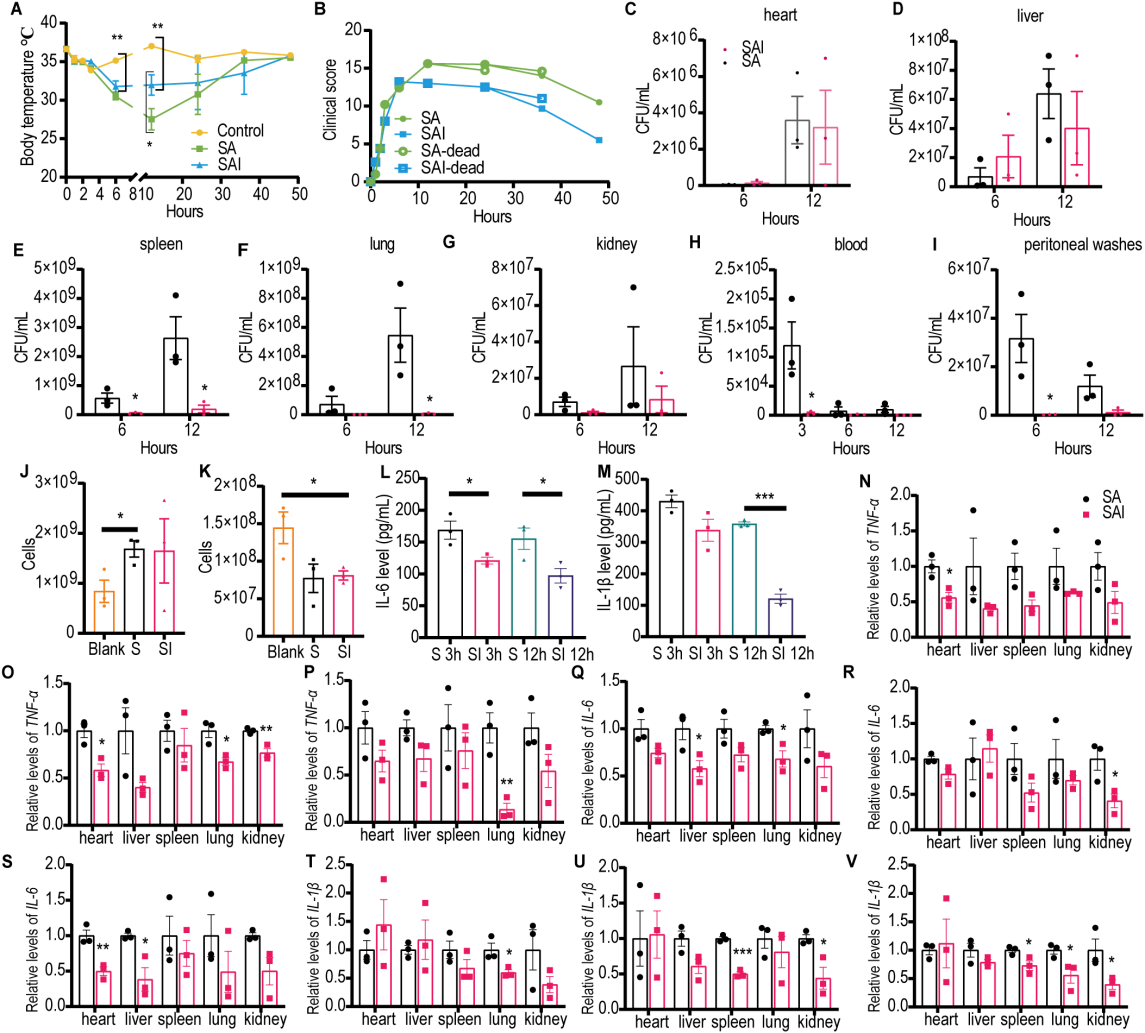
Irisin improved the performance of mice with SA induced septicemia. **(A)** Irisin increased body temperature in mice with SA sepsis. At each time point, the temperature of three mice in each group was measured. **(B)** Clinical manifestations of mice with SA sepsis improved by irisin. At each time point, data were collected from three mice in each group. **(C-I)** Bacterial load in organs (heart, liver, spleen, lung, kidney) and body fluids (blood and peritoneal washes) of mice with SA septicemia. n=3/group/time point. **(J)** Neutrophils in the blood of mice with septicemia induced by SA. n=3/group. **(K)** Monocytes in the blood of mice with septicemia induced by SA. n=3/group. **(L)** Serum IL-6 levels in mice with SA induced sepsis. n=3/group/time point. **(M)** Serum IL-1β levels in mice with SA induced sepsis. n=3/group/time point. **(N-P)** *TNF-α* mRNA levels in organs of mice at 3h, 6h and 12h of SA induced sepsis. n=3/group. **(Q-S)** *IL-6* mRNA levels in organs of mice at 3h, 6h and 12h of SA induced sepsis. n=3/group. **(T-V)** *IL-1β* mRNA levels in organs of mice at 3h, 6h and 12h of SA induced sepsis. n=3/group. Mean ± SEM, *P<0.05, **P<0.01, ***P<0.001.

To investigate the mechanism by which irisin improves the survival rate of mice with SA sepsis, BALB/c mice were challenged with 200 µL of SA at a concentration of 5×10^10^ CFU/mL, with or without an intraperitoneal injection of 5 μg/g irisin. As illustrated in **Figure 3A** and **Supplementary Table S4**, the body temperature of the control group remained stable within the normal range throughout the 48 h experiment. In contrast, the body temperature of mice in the *Staphylococcus aureus* CMCC 26003+irisin (SAI) group decreased from 36.64 ± 0.11°C to 31.78 ± 0.73°C during the first 6 h but subsequently increased to 35.76 ± 0.17°C by 48 h. Meanwhile, the body temperature of the SA group dropped from 36.62 ± 0.22°C to 27.52 ± 1.39°C in the initial 12 h, before rising to 35.5 ± 0.17°C over the next 36 h. Notably, the body temperature in the SAI group was significantly higher than that in the SA group at the 12 h mark, although it remained significantly lower than that of the control group at 6 and 12 h.

We assessed the effects of irisin on clinical manifestations—including appearance, behavior, respiratory characteristics, and hydration status—in SA septic mice and scored the results accordingly. As demonstrated in **Figure 3B** and **Supplementary Table S5**, the clinical scores for the SA group continued to escalate following SA challenge, peaking at 15.6 at 12 h, followed by a decline to 10.5 at 48 h. Conversely, clinical scores in the SAI group also increased after the SA challenge, reaching a maximum of 13.20 at 6 h, before decreasing to 5.50 at 48 h. At all observed time points post 12 h of SA challenge, the clinical scores in the SA group were consistently higher than those in the SAI group.

To assess bacterial load in SA septic mice, we conducted bacterial cultures on organ homogenates and body fluids from the mice. As shown in **Figures 3C–J** and **Supplementary Figures S2A–C**, 6 h post-infection, the viable bacterial count in the spleen of the SA group was significantly higher than that in the SAI group. At 12 h following infection, the viable bacteria count remained significantly elevated in both the spleen and lungs of the SA group compared to the SAI group. Additionally, in the blood, the number of viable bacteria in the SA group was significantly greater than that in the SAI group at 3 h post-infection, whereas peritoneal flushing solution also revealed significantly higher counts in the SA group at 6 h after infection. These findings suggest that irisin treatment may effectively reduce bacterial load in mice with SA sepsis.

The results of blood NEU detection indicated that the number of NEU in the SA group was significantly higher than that in the control group; however, no significant difference was observed between the SA group and the SAI group (**Figure 3K**). As illustrated in **Figure 3L**, blood MON detection revealed that the MON count in the SAI group was significantly elevated compared to the control group, with no significant difference noted between the SA and SAI groups. Furthermore, we assessed the pro-inflammatory factors IL-6 and IL-1β in the serum. At 3 h and 12 h post-bacterial challenge, the IL-6 concentration in the SAI group was significantly lower than that in the SA group (**Figure 3M**). Additionally, the IL-1β levels in the SAI group were significantly reduced compared to the SA group at 12 h after the bacterial attack, with no significant difference observed at 3 h (**Figure 3N**). Although irisin did not influence the counts of NEU and MON in the blood, it was found to decrease the levels of inflammatory factors in the serum. These findings suggest that irisin plays a role in the progression of SA sepsis.

We also investigated the effects of irisin on the transcription levels of pro-inflammatory factors *TNF-α*, *IL-6*, and *IL-1β* across various organs in SA septic mice. As shown in **Figures 3N–V**, at least one pro-inflammatory transcription factor in the SAI group was significantly down-regulated in the heart, liver, spleen, lung, and kidney within 12 h following bacterial exposure, except for the spleen at 3 h, kidney at 3 h, and liver at 6 h. These results imply that irisin contributes to reducing inflammation in the organs of SA septic mice.

### Irisin enhanced the phagocytosis of splenic macrophages in septicemic mice

The phagocytic capability of spleen macrophages is crucial for eliminating pathogenic microorganisms from the bloodstream, establishing their vital role in the body’s defense against sepsis. In this experiment, EGFP was constructed. The plasmid and bacterial strain are shown in **Supplementary Figures S3A, B**. Sepsis was induced in mice with 1×10^9^ CFU/mL EGFP, and treatment with 5 μg/g irisin increased the survival rate from 33.3% to 83.3% (**Supplementary Figure S3E**).

**Figure 4:**
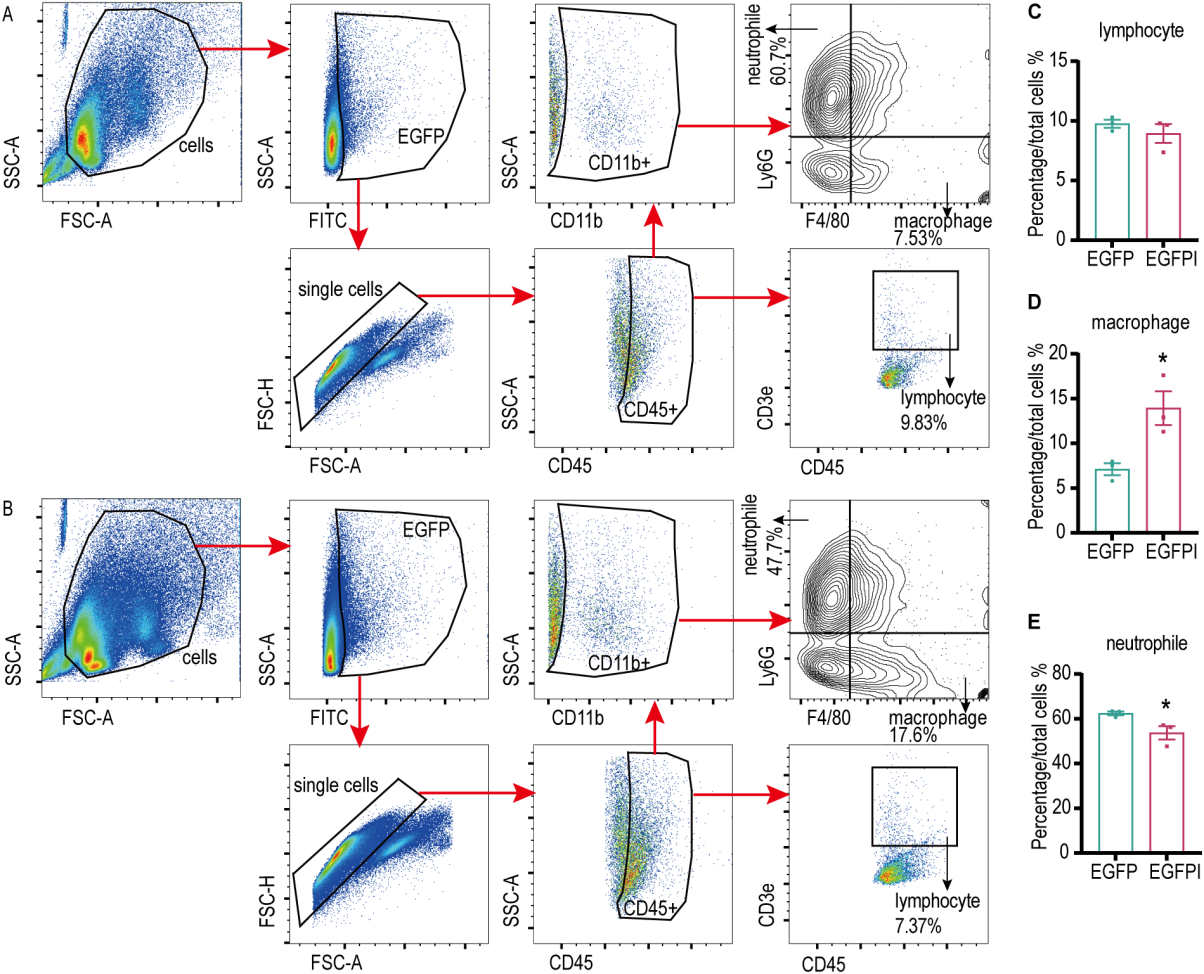
Irisin enhanced the phagocytosis of splenic macrophages in septicemic mice. **(A)** Phagocytosis of EGFP by splenic immune cells of septicemic mice. **(B)** Irisin enhanced phagocytosis of EGFP in splenic immune cells of septicemic mice. **(C-E)** Statistical results of phagocytosis by lymphocytes, macrophages and neutrophils. n=3/group. Mean ± SEM, *P<0.05, **P<0.01, ***P<0.001.

**Figure 5:**
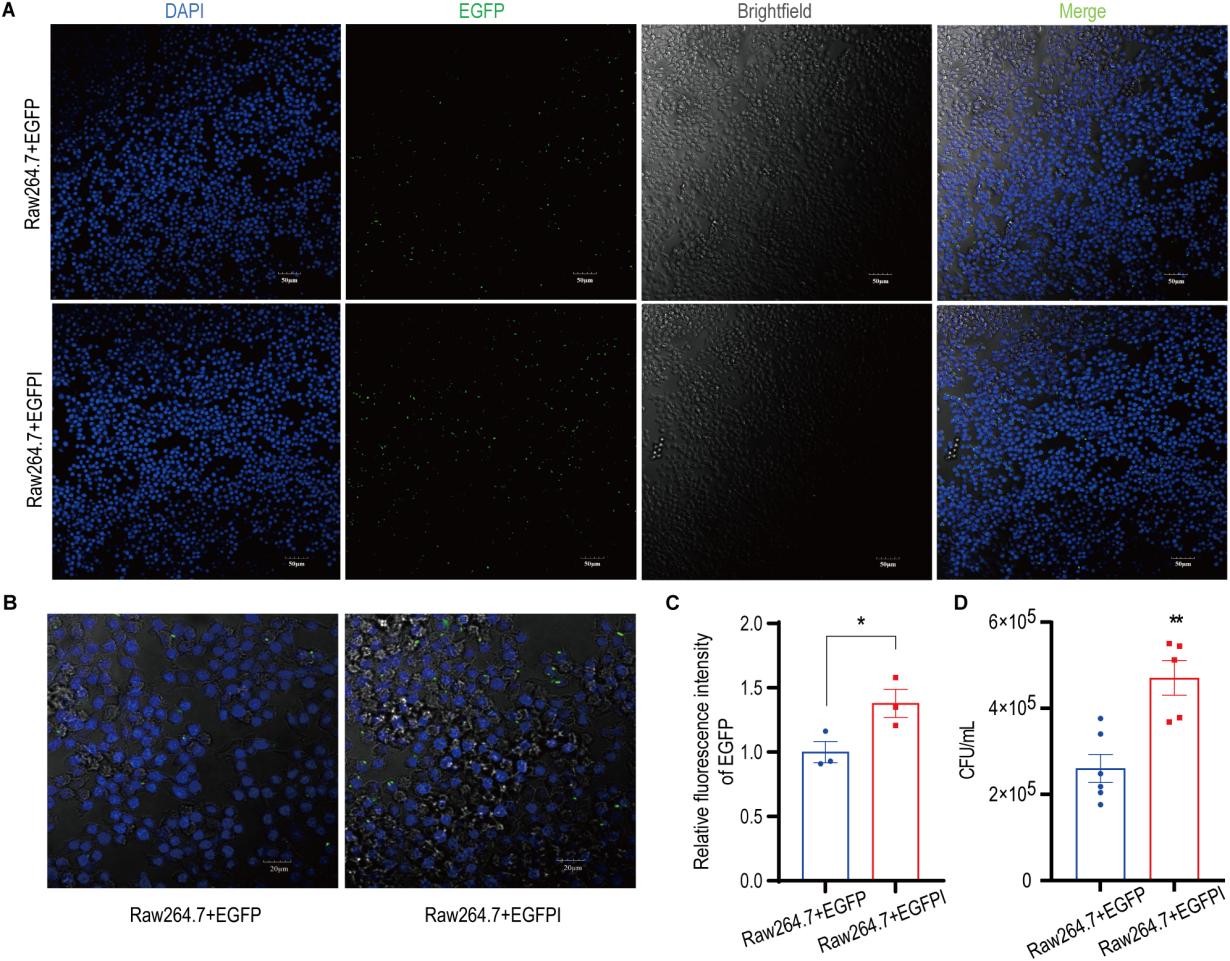
Irisin enhanced the phagocytosis of macrophages. **(A)** Irisin promoted phagocytosis of EGFP (green) by RAW 264.7 cells. DAPI (blue), bar=50μm. **(B)** Irisin promoted phagocytosis of EGFP (green) by RAW 264.7 cells. DAPI (blue), bar=20μm. **(C)** Phagocytosis (relative fluorescence intensity) statistics of EGFP by RAW 264.7 cells in **(A)**. **(D)** Irisin promoted phagocytosis of EGFP by RAW 264.7 cells (Live bacteria count). Mean ± SEM, *P<0.05, **P<0.01, ***P<0.001.

To evaluate the effect of irisin on the phagocytosis activity of spleen macrophages, spleen macrophages were collected from EGFP septicemic mice 24h after bacterial infection and analyzed using flow cytometry. As shown in **Figures 4A–E** and **Supplementary Figure S4A–D**, compared to the EGFP group, EGFPI group demonstrated a significant reduction in neutrophils containing EGFP in the spleen, accompanied by a notable increase in macrophage numbers. To further investigate the effect of irisin on macrophage phagocytosis, *in vitro* phagocytosis assays were performed using RAW 264.7 cells. The results of fluorescence signal analysis indicated that the phagocytosis of RAW 264.7 cells against EGFP was significantly enhanced following treatment with 20 nM irisin (**Figures 5A–C**). Additionally, bacterial count results confirmed that the phagocytosis of RAW 264.7 cells against EGFP significantly increased after incubation with 20 nM irisin (**Figure 5D**). These findings suggest that irisin enhances the phagocytosis of macrophages against bacteria in both *in vivo* and *in vitro* settings.

### Irisin increased the proliferative activity and anti-inflammatory ability of macrophages

**Figure 6:**
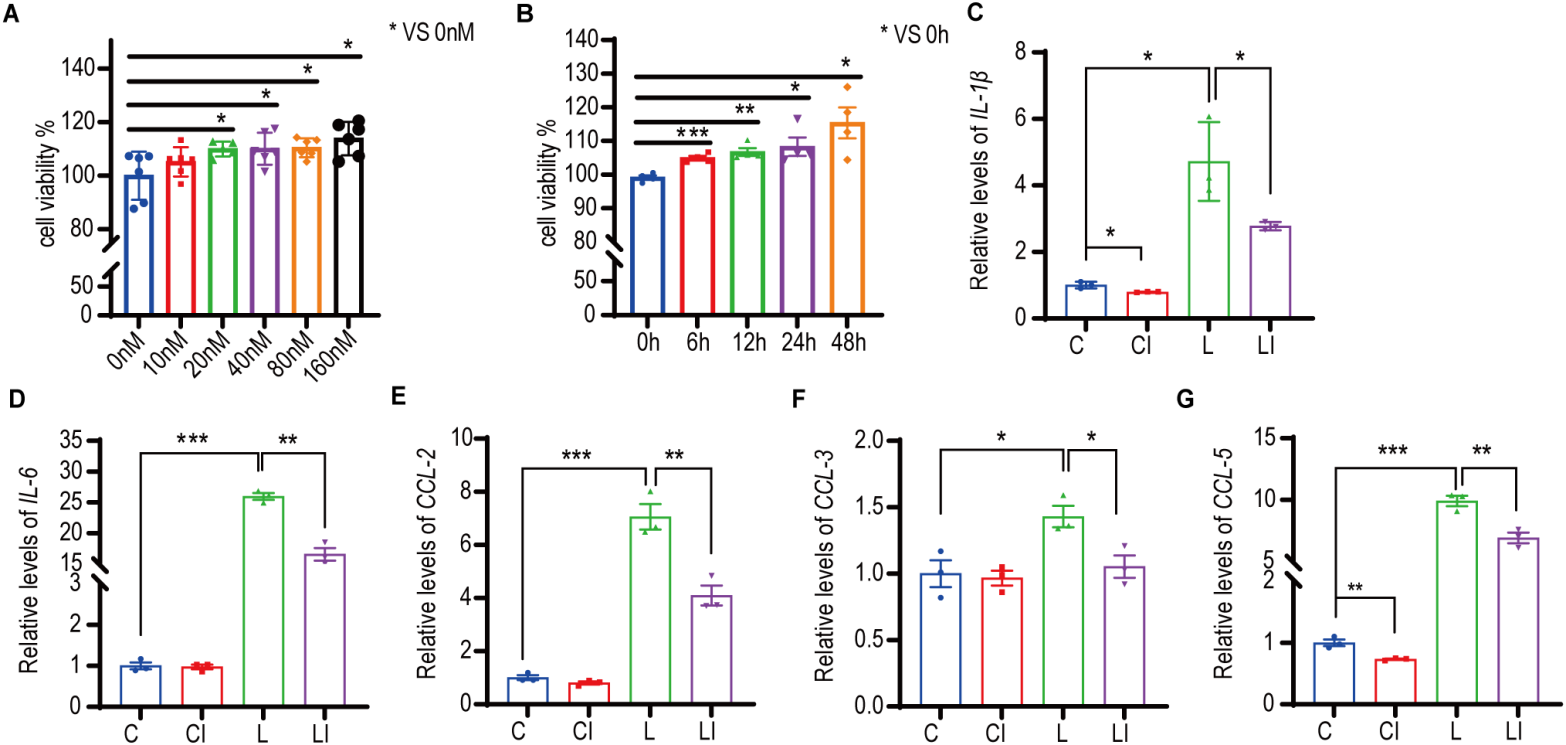
Irisin increased the proliferative activity and anti-inflammatory ability of macrophages. **(A)** Effects of 0-160nM irisin on the proliferation activity of RAW 264.7 cells for 12h. n=5 or 6 in each group. *P<0.05. **(B)** Effects of 20nM irisin treatment for 0-48h on the proliferation activity of RAW 264.7 cells. n=4 in each group. **(C-G)** 20nM irisin reduced *IL-1β*, *IL-6*, *CCL-2*, *CCL-3* and *CCL-5* mRNA levels of LPS-induced inflammation in RAW264.7 cells. n=3/group. Mean ± SEM, *P<0.05, **P<0.01, ***P<0.001.

RAW 264.7 cells were treated with concentrations of 0-160 nM irisin for 12 h, and cell proliferation activity was measured using the CCK-8 assay. As illustrated in **Figure 6A**, cell proliferation activity significantly increased with irisin concentrations ranging from 20 nM to 160 nM. Subsequently, RAW 264.7 cells were treated with 20 nM irisin, and cell proliferation activity was measured via CCK-8 within 0-48 h. The results presented in **Figure 6B** demonstrate a significant increase in cell proliferation activity at intervals from 6 to 48 h.

We developed a RAW 264.7 cell inflammation model to explore the effects of irisin on cellular inflammation. **Figures 6C, D** show that, following LPS treatment of RAW 264.7 cells, the relative transcription levels of the inflammatory factors *IL-1β* and *IL-6* increased significantly, indicating the successful establishment of the inflammatory model. After treatment with 20 nM irisin, the relative transcription levels of *IL-1β*, *IL-6*, C-C motif ligand 2 (*CCL2*), C-C motif ligand 3 (*CCL3*), and C-C motif ligand 5 (*CCL*5) in the LPS+irisin (LI) group were significantly decreased compared to the LPS (L) group (**Figures 6C–G**). Similarly, in comparison to the control (C) group, the relative transcription levels of *IL-1β* and *CCL5* were significantly lower in the control+irisin (CI) group (**Figures 6C, G**). These results suggest that irisin can effectively reduce inflammation levels in RAW 264.7 cells.

Our previous publications reported that irisin was recognized by 3T3-L1 cells through integrin αV (33). We conducted similar experiments using RAW 264.7 cells. As illustrated in **Supplementary Figures S5A**, the irisin group exhibited both irisin-positive and integrin αV-positive reactions in RAW 264.7 cells. Following the combination of fluorescence channels, a broad range of double-positive areas was observed. Notably, fluorescence results in the control group, which comprised RAW 264.7 cells not treated with irisin, also displayed a positive reaction for the irisin antibody (**Supplementary Figures S5B**). The negative control group, which involved the use of an unincubated fluorescent secondary antibody, indicated a negative reaction in the irisin and integrin αV fluorescent channels, ruling out the possibility of interference from spontaneous cell fluorescence (**Supplementary Figure S5A**). These results indicate that RAW 264.7 cells in the control group spontaneously produced irisin, which was recognized by integrin αV. Additionally, Western blot analysis demonstrated that RAW 264.7 cells could synthesize FNDC5 protein, the precursor of irisin (**Supplementary Figure S5C**). Further analysis confirmed that RAW 264.7 cells produced irisin protein (**Supplementary Figure S5D**). Collectively, these findings suggest that RAW 264.7 cells recognize irisin through integrin αV and are capable of producing irisin.

## Discussion

The data from this study demonstrate that intraperitoneal injection of irisin in mice alleviates sepsis by enhancing the phagocytosis of spleen macrophages against bacteria and inhibiting inflammation.

Sepsis poses a significant threat to human life (1), occurring when the body’s barrier tissues are compromised by bacterial infection (1, 3–5). Previous research reported that serum irisin levels in sepsis patients (12.85 ± 0.41 ng/mL) were markedly reduced compared to healthy individuals (16.35 ± 0.58 ng/mL) (33). We speculate that a similar phenomenon occurs in mice with sepsis. To test this hypothesis, a bacterial induction approach was employed to establish the septic mice model. The results revealed that serum irisin levels in E septicemia mice (11.24 ± 1.33 ng/mL) and SA septicemia mice (4.52 ± 0.87 ng/mL) were significantly lower than those in healthy mice (40.02 ± 1.17 ng/mL), thereby confirming our hypothesis.

Irisin treatment has been shown to reduce acute kidney injury and suppress ferroptosis in the liver of mice with sepsis (33, 36). However, few studies have examined the physical signs of the mice, which limits the ability to directly assess the therapeutic effects of irisin on sepsis. Our findings indicate that a treatment of 5 μg/g irisin significantly improved the survival rates of mice with E sepsis (from 16.7% to 66.7%) and those with SA sepsis (from 40% to 80%). Furthermore, a lower dose of 0.5 μg/g irisin also enhanced survival rates in septic mice, although its efficacy was not as pronounced as that of the 5 μg/g dosage. Notably, all mice treated with 50 μg/g irisin succumbed during the study period; however, this dosage did not affect the survival rate of healthy mice (**Supplementary Figure S6A**). Previous research has reported the administration of irisin at doses of 0.5 μg/kg/day (37), 250 μg/kg (36, 38), and 500 μg/kg/day (39) without resulting in mortality. These findings, along with our results, suggest that the deaths of the mice with sepsis were not attributable to irisin treatment. According to our earlier research (35), irisin can upregulate uncoupling protein 1 (UCP-1) and promote thermogenesis. We hypothesize that the 50 μg/g irisin treatment may exacerbate metabolic disorders in septic mice, leading to accelerated energy consumption and an inability to maintain body temperature, ultimately resulting in mortality. However, the underlying mechanisms warrant further investigation.

A treatment with 5 μg/g of irisin in mice suffering from septicemia has been shown to slow the drop in body temperature and expedite temperature recovery. Moreover, it improved clinical manifestations, reduced bacterial load in organs and body fluids, and lowered the levels of inflammatory factors in serum, thus decreasing inflammation levels within the organs. Notably, as sepsis progressed, the bacterial load in each organ increased; however, irisin treatment effectively reduced this bacterial burden. These findings suggest that irisin may enhance the body*’*s ability to resist bacterial invasion. The number of viable bacteria in both blood and peritoneal flush decreased progressively during the advancement of sepsis, and treatment with irisin accelerated this elimination process, indicating that irisin facilitates the clearance of bacteria from body fluids. Neutrophils, key innate immune cells combating pathogens (40), were elevated in both septic and irisin-treated groups compared to controls, with no significant difference between septic and irisin groups. Monocytes, which migrate to inflammatory sites differentiating into macrophages/dendritic cells (41), decreased in septic and irisin-treated groups versus controls, but showed no intergroup difference. Consistent results across E and SA septic models collectively indicate that irisin neither suppresses neutrophil-mediated innate immunity nor significantly affects circulating monocyte recruitment. Overall, these results provide clear evidence of the mitigating effects of irisin on the progression of sepsis.

The spleen is a critical peripheral immune organ in the body, playing a significant role in the defense against infections, particularly sepsis. The phagocytosis conducted by splenic macrophages is vital for eliminating pathogenic microorganisms from the bloodstream. In patients who have undergone splenectomy, the ability of monocytes to phagocytize and eliminate Candida albicans is notably diminished, which correlates with an increased susceptibility to sepsis (15). Furthermore, a study revealed that Salmonella present in the peripheral blood of typhoid patients is ultimately engulfed by splenic macrophages (16). In canines, absence of the phagocytosis mechanism following splenectomy results in reduced clearance of endotoxins (17). These findings suggest a direct role of the spleen in the clearance of bacteria during sepsis. Phagocytosis by macrophages and neutrophils represents the initial response of the body’s immune system to bacterial invasion. The sepsis ensuing from bacterial infections across multiple organs leads to recruitment of circulating monocytes to the infection sites, where they differentiate into macrophages. These macrophages are responsible for eliminating foreign substances, as well as aged and damaged cells, thereby maintaining the body’s internal homeostasis (42, 43). Neutrophils exhibit a potent bactericidal effect due to the presence of neutral proteins and oxidants in their cytoplasm, which results in significant cytotoxicity (44, 45). A substantial influx of neutrophils occurs at the site of infection, and the timely clearance of senescent neutrophils by macrophages is crucial for the body’s recovery. Our results have indicated that irisin treatment leads to a decrease in the proportion of neutrophils phagocytizing EGFP in the spleen. However, the survival rate of mice with E and SA sepsis improved following irisin treatment, accompanied by a reduction in clinical symptoms, suggesting that the bactericidal capacity of the spleen remained intact. This implies that the decreased neutrophil population was primarily composed of senescent neutrophils. Additionally, prior studies have demonstrated that phagocytosis of neutrophil components enhances the antibacterial activity of macrophages against Candida albicans (46) and Mycobacterium tuberculosis (47) *in vitro*. Therefore, we propose that irisin treatment enhances the phagocytic capacity of macrophages in the spleen, enhancing the clearance of aged neutrophils and ultimately improving bacterial phagocytosis by these macrophages. Phagocytosis assays conducted on RAW 264.7 cells revealed that irisin treatment significantly improved the phagocytic activity of these cells towards EGFP. Overall, these results indicate that irisin reduces the bacterial load in the organs of septicemic mice by enhancing the phagocytotic function of splenic macrophages.

Sepsis leads to widespread inflammation in the body and exacerbates the dysfunction of various organs. Therefore, suppressing inflammation could help alleviate sepsis. Our findings demonstrate that irisin, at concentrations ranging from 20 nM to 160 nM, significantly enhances the proliferation of RAW 264.7 cells within 12 h. Specifically, treatment with 20 nM irisin increased the proliferation activity of these cells between 6 and 48 h. Previous studies (48) have indicated that incubation of RAW 264.7 cells with 50 nM and 100 nM irisin for 24 h improves cell proliferation. Our results expand on this knowledge by demonstrating the promoting effect of irisin on RAW 264.7 cell proliferation across a broader dose and time range. To simulate inflammation, we treated RAW 264.7 cells with LPS alongside 20 nM irisin therapy. The results revealed that irisin treatment reduced mRNA levels of *IL-1β* and *IL-6*, suggesting that 20 nM irisin treatment may alleviate inflammation in RAW 264.7 cells. Consistent with this, Mazur-Bialy (31) reported that treatment with 50 nM and 100 nM irisin reduces *IL-1β* and *IL-6* mRNA levels in inflamed RAW cells; our findings achieve similar results at lower concentrations of irisin. Additionally, 20 nM irisin was observed to decrease mRNA levels of *IL-1β* and *CCL-5* in RAW 264.7 cells, indicating its ability to inhibit inflammation. Our previous reports (35) showed that irisin is recognized by adipocytes via integrin αv, and recent results confirm that it is also recognized by RAW 264.7 cells through integrin αv. Furthermore, RAW 264.7 cells were identified as a source of irisin, providing insight into its origin and specific receptor.

A limitation of this study was that the treatment was monitored over a relatively short duration. Sepsis often arises as a complication of other diseases, which can be difficult to detect and treat promptly. Although irisin treatment alleviated sepsis in mice and inhibited inflammation in RAW 264.7 cells within a short timeframe, it remains essential to evaluate the therapeutic effects of irisin over a longer period. Although current results indicate the potential efficacy of irisin for sepsis, long-term evaluation should be the focus of future research.

In conclusion, sepsis reduces serum irisin levels in mice, and intraperitoneal injections of irisin mitigate the effects of sepsis. This benefit is achieved through the enhancement of phagocytosis in spleen macrophages against bacteria, along with the inhibition of inflammation.

## Data Availability

The original contributions presented in the study are included in the article/**Supplementary Material**. Further inquiries can be directed to the corresponding author.
