# Supplementary material for: Irisin alleviated sepsis via enhancing macrophage phagocytosis and reducing inflammation levels
Source: Front Immunol. 2025 Aug 15;16:1618699. doi: 10.3389/fimmu.2025.1618699 (PMC12394160; doi:10.3389/fimmu.2025.1618699)
Supplement: Supplementary file 1 [file DataSheet1.pdf]

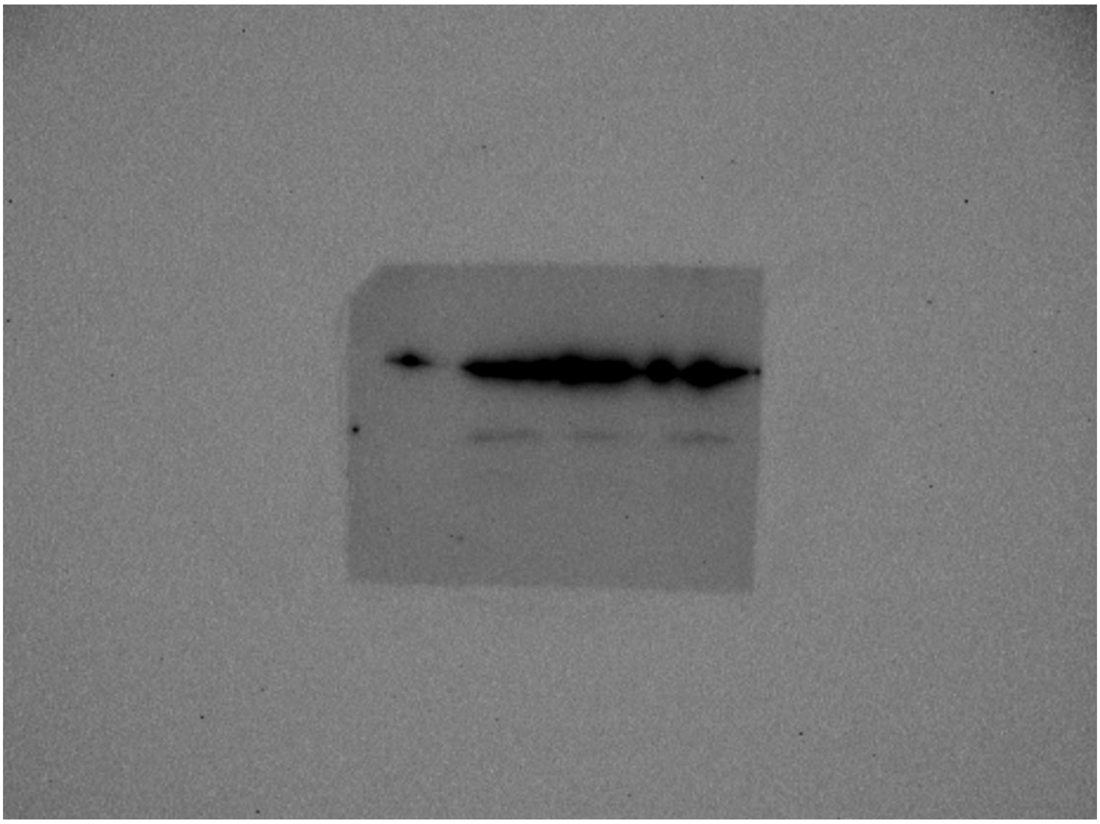

Full unedited gel for Figure S5 (C). FNDC5 protein was produced in RAW 264.7 cells.

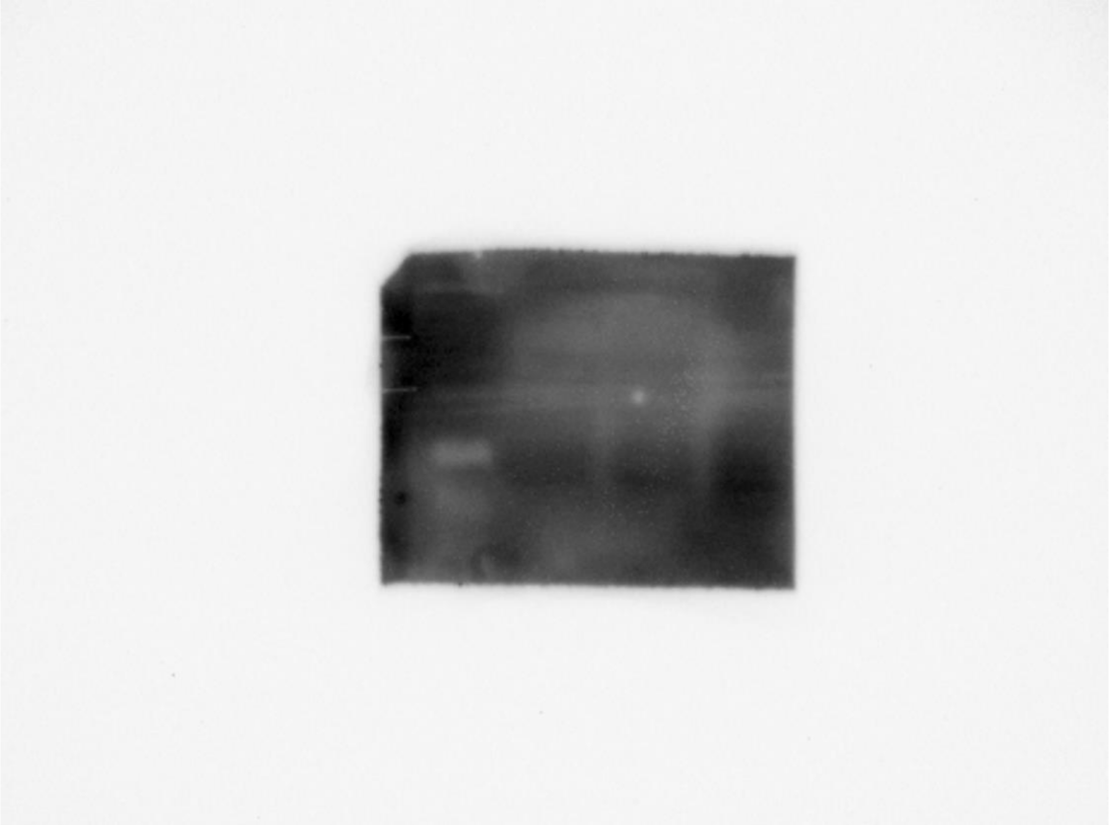

Full unedited gel for Figure S5 (D). Irisin protein was produced in RAW 264.7 cells.
