# Supplementary material for: Irisin alleviated sepsis via enhancing macrophage phagocytosis and reducing inflammation levels
Source: Front Immunol. 2025 Aug 15;16:1618699. doi: 10.3389/fimmu.2025.1618699 (PMC12394160; doi:10.3389/fimmu.2025.1618699)
Supplement: Supplementary file 2 [file Presentation1.pdf]

Supplemental Table S1

Table S1. Body temperature of mice with E sepsis

	control	E	EI
0h	36.73±0.20	36.73±0.14	36.46±0.17
1h	35.6±0.28	34.7±0.17	34.96±0.23
2h	35.96±0.13	34.63±0.18	35.16±0.03
3h	36.1±0.45	33.83±0.23	34.26±0.37
6h	35.2±0.15	30.73±0.08	31.5±0.20
12h	36.2±0.15	30.1±0.32	30.63±0.20
24h	36.9±0.17	29.5±0.45	32.7±0.41
36h	35.4±0.23	28.5±0.51	34.03±0.58
48h	36.03±0.08	30.66±1.27	34.13±0.44

Supplemental Table S2

Table S2. Standard used for assigning clinical scores (34)

Observing targets	condition	score
appearance	normal	0
	lack of grooming	1
	piloerection	2
	hunched up	3
	above and eyes half closed	4
behaviour - unprovoked	normal	0
	minor changes	1
	less mobil and flock together	2
	restless or very still	3
behaviour - provoked	responsive and alert	0
	unresponsive and not alert	3
clinical signs	normal respiratory rate	0
	slight changes	1
	decreased rate with abdominal breathing	2
	tachypnea and cyanosis	3
hydration status	normal	0
	dehydrated	5

Supplemental Table S3

Table S2. Average clinical scores of mice with E sepsis

	E	EI
0h	0.00	0.00
1h	7.83	6.50
2h	8.00	7.17
3h	10.17	8.50
6h	14.50	10.50
12h	17.17	14.50
24h	17.67	14.00
36h	17.00	10.00
48h	11.00	7.25

Supplemental Table S4

Table S4. Body temperature of mice with E sepsis

	control	SA	SAI
0h	36.64±0.16	36.62±0.22	36.64±0.11
1h	35.34±0.43	35.04±0.15	34.98±0.2
2h	35.1±0.28	34.94±0.15	35.16±0.25
3h	33.9±0.37	34.14±0.07	35.04±0.3
6h	35.14±0.36	30.5±0.45	31.78±0.73
12h	37.02±0.16	27.52±1.39	31.98±1.32
24h	35.38±0.46	30.75±2.62	32.25±3.47
36h	36.24±0.17	35.16±0.17	33.53±2.76
48h	35.82±0.13	35.5±0.17	35.76±0.17

Supplemental Table S5

Table S5. Average clinical scores of mice with SA sepsis

	SA	SAI
0h	0.00	0.00
1h	1.00	2.60
2h	4.40	4.40
3h	10.20	8.00
6h	12.40	13.20
12h	15.60	13.00
24h	15.50	12.50
36h	14.00	9.63
48h	10.50	5.50

Supplemental Table S6

Table S6. List of qRT-PCR primers.

Speices	gene	Forward	Reverse
mouse	IL-1β	TGCCACCTTTTGACAGTGATG	TTCTTGTGACCCTGAGCGAC
mouse	IL-6	GGAAATCGTGGAAATGAG	CCAGAAGACCAGAGGAAA
mouse	TNF-α	ACCGTCAGCCGATTTGCTAT	TTGGGCAGATTGACCTCAGC
mouse	CCL2	TGGGTCCAGACATACATT	ACGGGTCAACTTCACATT
mouse	CCL3	ACTGACCTGGAACTGAATG	GAAGAGTCCCTCGATGTG
mouse	CCL5	CCACTCCCTGCTGCTTTG	CACTTGGCGGTTCCTTCG
mouse	β-actin	TTGCTGACAGGATGCAGAAG	ACATCTGCTGGAAGGTGGAC

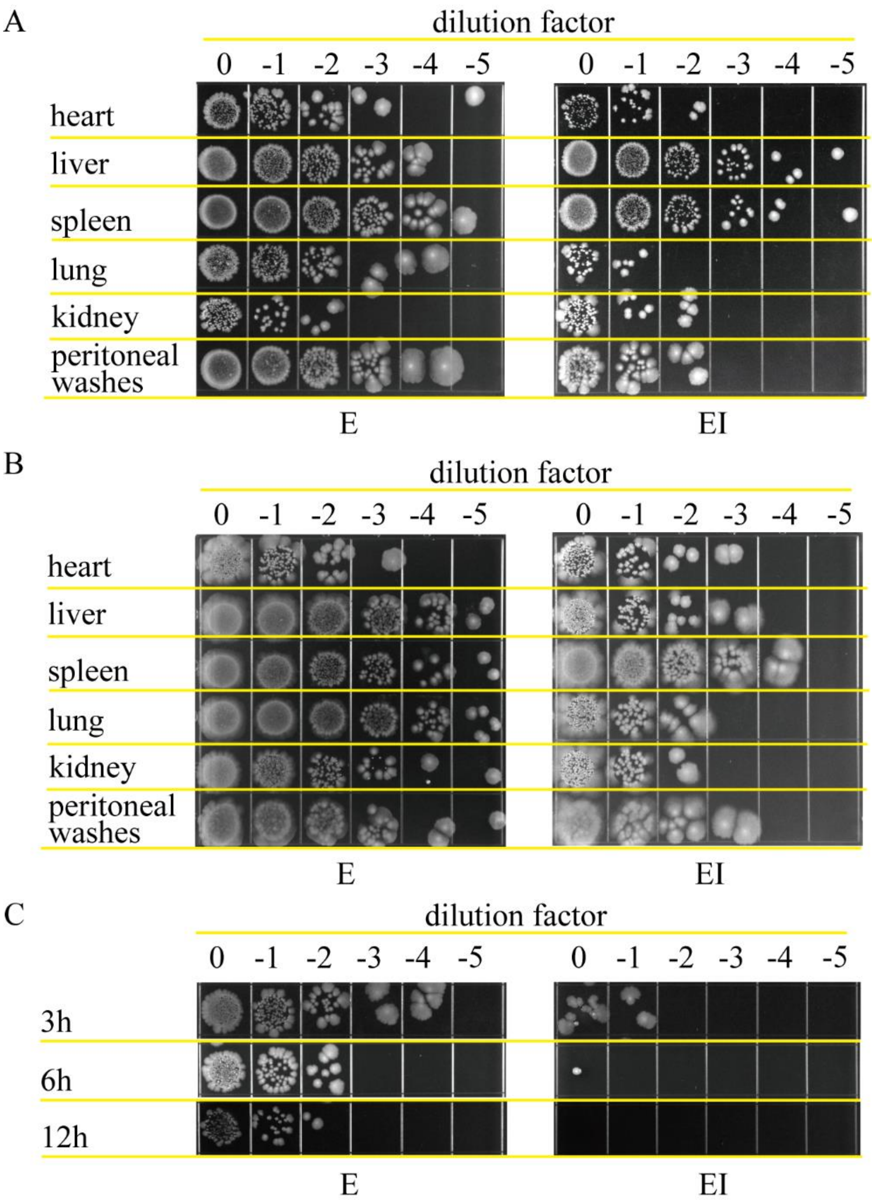

Figure S1 Bacterial load in organs and body fluids of mice with E septicemia.

(A) Bacterial load in heart, liver, spleen, lung, kidney and blood of mice with E septicemia at 6h following the bacterial attack. n=3/group. (B) Bacterial load in heart, liver, spleen, lung, kidney and blood of mice with E septicemia at 12h following the bacterial attack. n=3/group. (C) Bacterial load in peritoneal washes of mice with E septicemia at 3h, 6h and 12h following the bacterial attack. n=3/group.

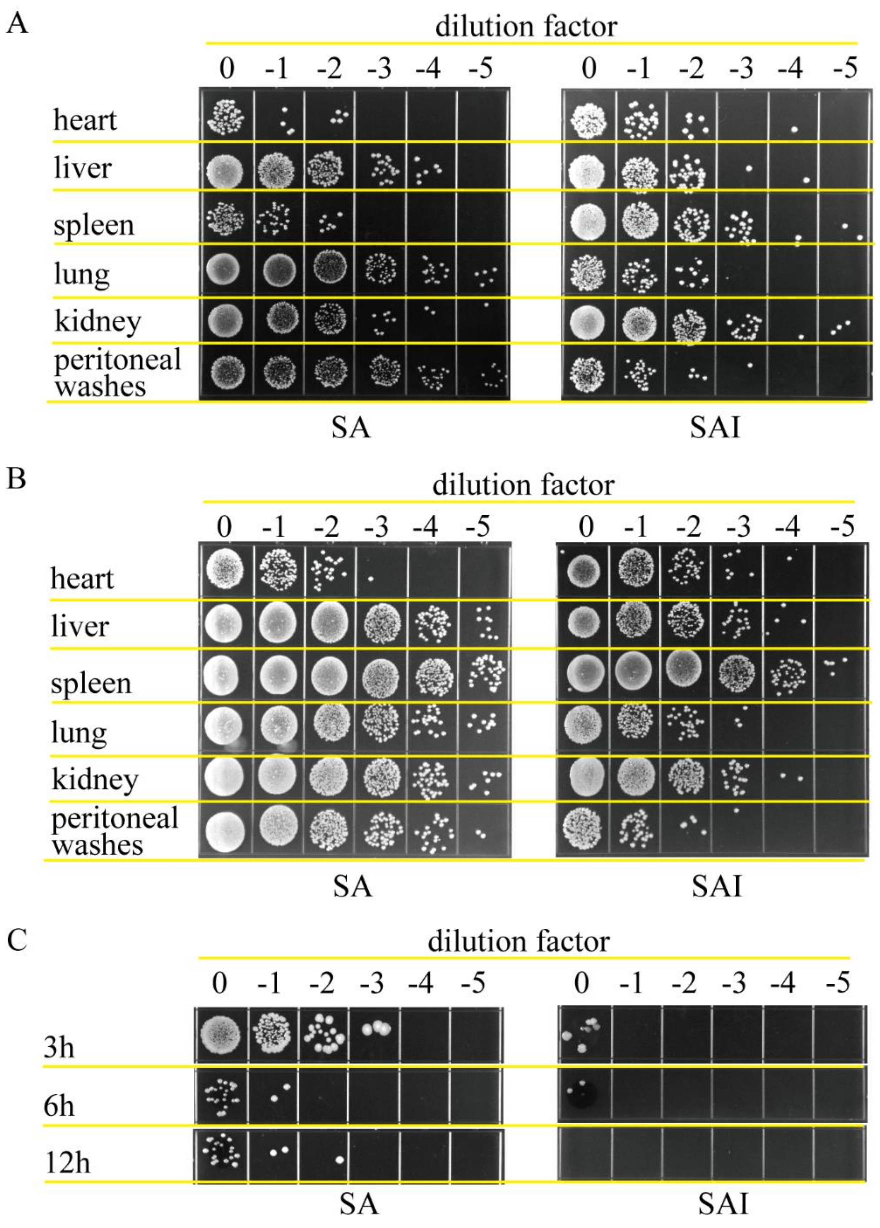

Figure S2 Bacterial load in organs and body fluids of mice with SA septicemia.

(A) Bacterial load in heart, liver, spleen, lung, kidney and blood of mice with SA septicemia at 6h following the bacterial attack. n=3/group. (B) Bacterial load in heart, liver, spleen, lung, kidney and blood of mice with SA septicemia at 12h following the bacterial attack. n=3/group. (C) Bacterial load in peritoneal washes of mice with SA septicemia at 3h, 6h and 12h following the bacterial attack. n=3/group.

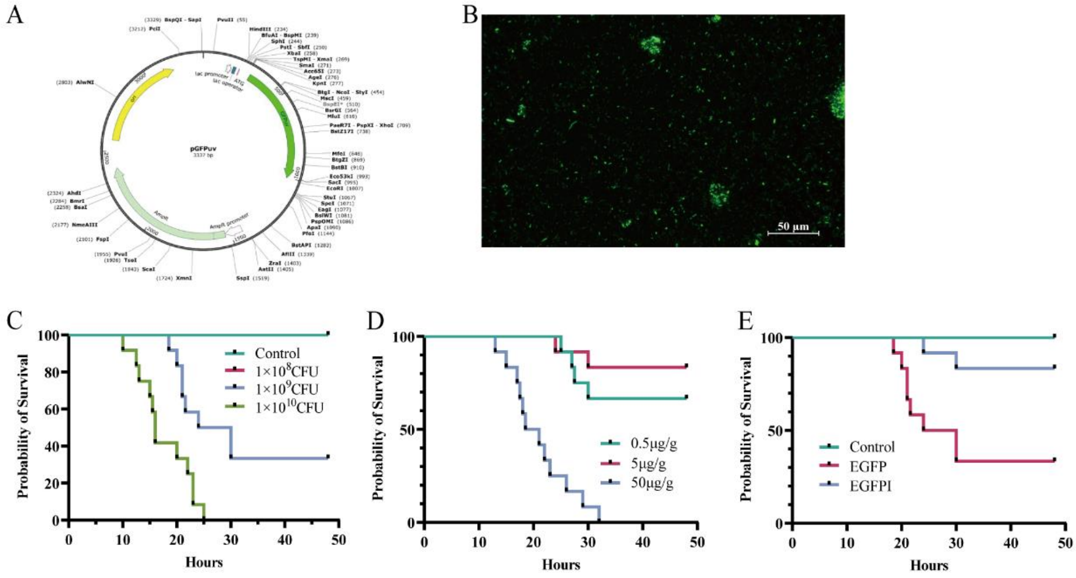

Figure S3 Irisin enhanced the survival rate of mice with EGFP septicemia.

(A) pGFPuv plasmid. (B) DH5α E. coli carrying pGFPuv plasmid. (C) Optimal dose of EGFP induced sepsis in mice. n=12/group. (D) Optimal dose of Irisin for relieving EGFP induced sepsis in mice. n=12/group. (E) The survival rate of mice with sepsis induced by EGFP improved by irisin. n=12/group.

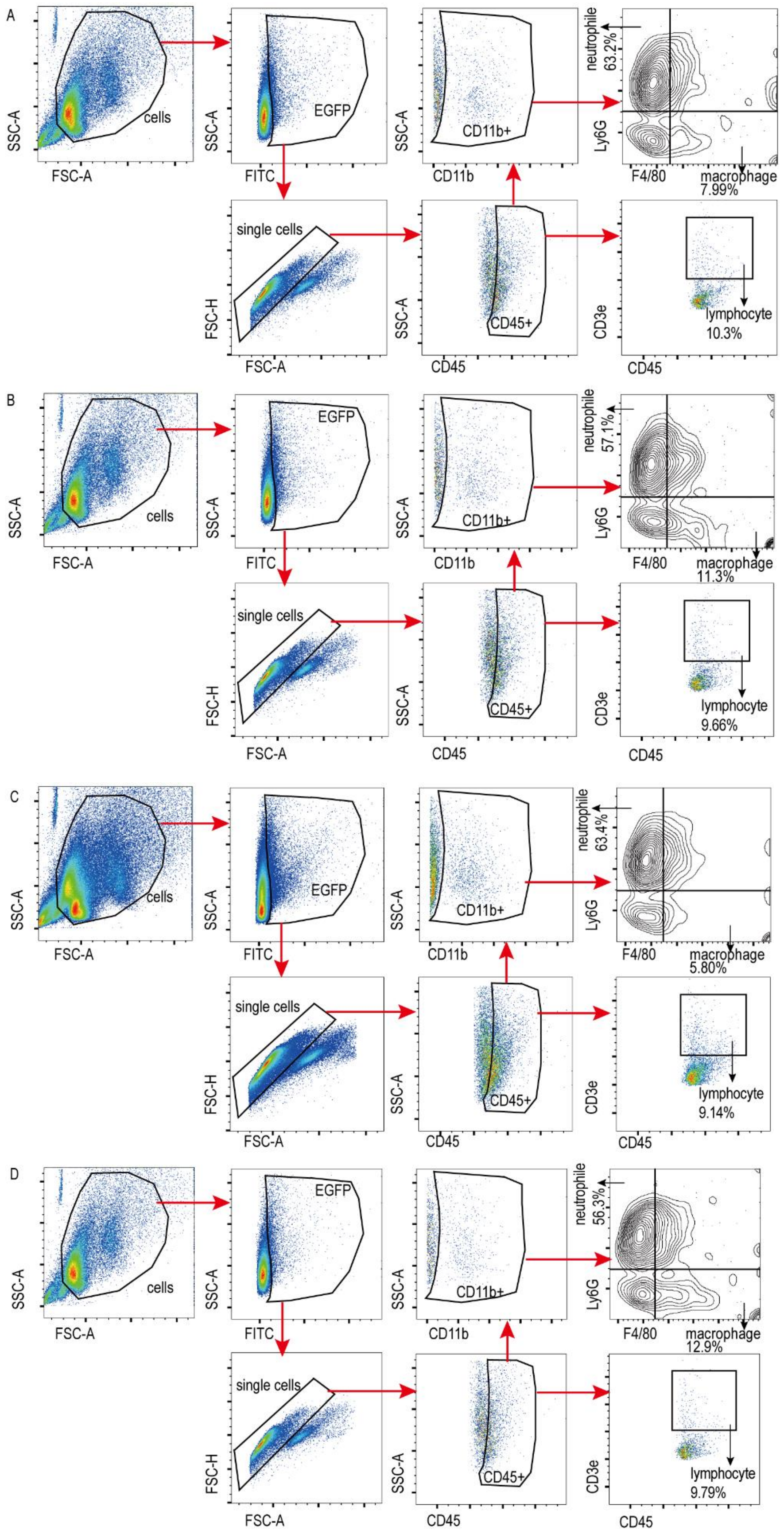

Figure S4 Irisin enhanced the phagocytosis of splenic macrophages in septicemic mice. (A), (C) Phagocytosis of EGFP by splenic immune cells of septicemic mice.

(B), (D) Irisin enhanced phagocytosis of EGFP in splenic immune cells of septicemic mice.

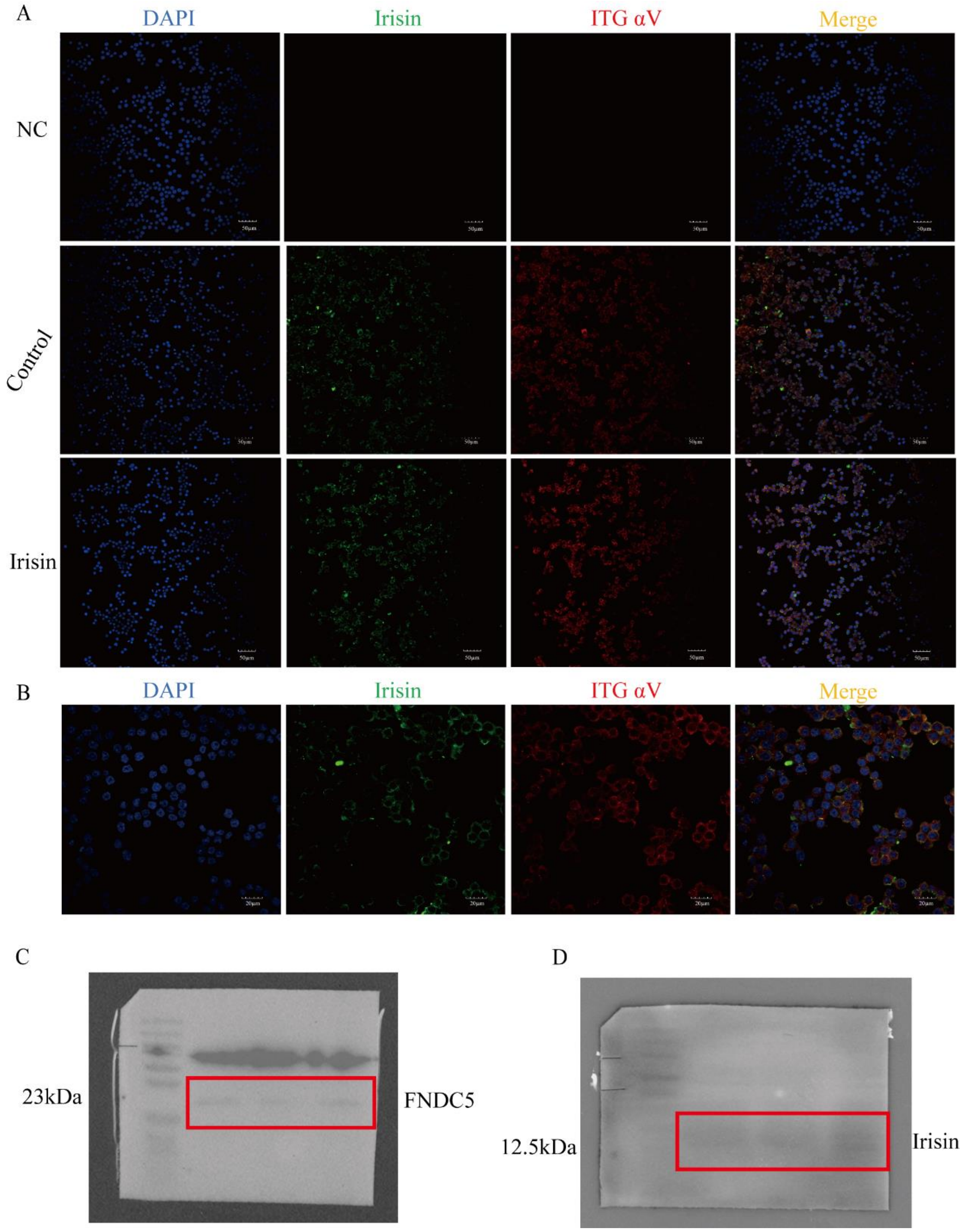

Figure S5 Irisin can be produced and recognized by RAW264.7 cells.

(A) Recognition of Irisin (green) by ITG αV (red) in RAW 264.7 cells. DAPI (blue), bar=50μm. (B) Recognition of Irisin (green) by ITG αV (red) in RAW 264.7 cells. DAPI (blue), bar=20μm. (C) FNDC5 protein was produced in RAW 264.7 cells. (D) Irisin protein was produced in RAW 264.7 cells.

A

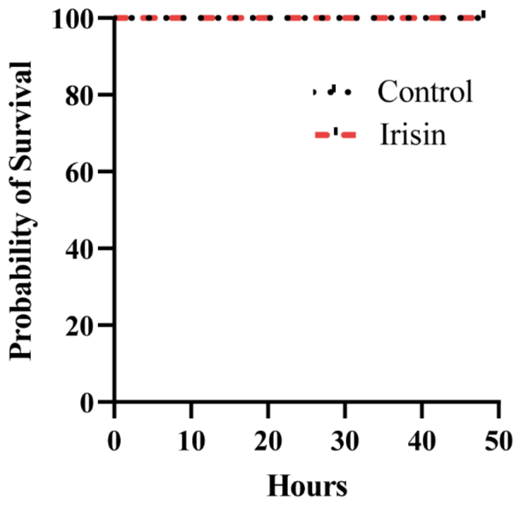

Figure S6 50 μg/g irisin did not kill the mice.

(A) Toxicity test of 50 μg/g irisin on mice, n=10/group.
